# Analysis of partial and complete protection in malaria cohort studies

**DOI:** 10.1186/1475-2875-12-355

**Published:** 2013-10-05

**Authors:** Matthew E Cairns, Kwaku Poku Asante, Seth Owusu-Agyei, Daniel Chandramohan, Brian M Greenwood, Paul J Milligan

**Affiliations:** 1 MRC Tropical Epidemiology Group, London School of Hygiene & Tropical Medicine, London, UK; 2 Kintampo Health Research Centre, Kintampo, Ghana; 3 Faculty of Infectious and Tropical Diseases, London School of Hygiene & Tropical Medicine, London, UK

**Keywords:** Malaria epidemiology, Heterogeneity, Overdispersion, Zero-inflation

## Abstract

**Background:**

Malaria transmission is highly heterogeneous and analysis of incidence data must account for this for correct statistical inference. Less widely appreciated is the occurrence of a large number of zero counts (children without a malaria episode) in malaria cohort studies. Zero-inflated regression methods provide one means of addressing this issue, and also allow risk factors providing complete and partial protection to be disentangled.

**Methods:**

Poisson, negative binomial (NB), zero-inflated Poisson (ZIP) and zero-inflated negative binomial (ZINB) regression models were fitted to data from two cohort studies of malaria in children in Ghana. Multivariate models were used to understand risk factors for elevated incidence of malaria and for remaining malaria-free, and to estimate the fraction of the population not at risk of malaria.

**Results:**

ZINB models, which account for both heterogeneity in individual risk and an unexposed sub-group within the population, provided the best fit to data in both cohorts. These approaches gave additional insight into the mechanism of factors influencing the incidence of malaria compared to simpler approaches, such as NB regression. For example, compared to urban areas, rural residence was found to both increase the incidence rate of malaria among exposed children, and increase the probability of being exposed. In Navrongo, 34% of urban residents were estimated to be at no risk, compared to 3% of rural residents. In Kintampo, 47% of urban residents and 13% of rural residents were estimated to be at no risk.

**Conclusion:**

These results illustrate the utility of zero-inflated regression methods for analysis of malaria cohort data that include a large number of zero counts. Specifically, these results suggest that interventions that reach mainly urban residents will have limited overall impact, since some urban residents are essentially at no risk, even in areas of high endemicity, such as in Ghana.

## Background

Malaria transmission is highly heterogeneous in endemic areas, with a small fraction of the population suffering a disproportionately large fraction of infections and clinical disease [1]. Recognition of the fact that a sub-group of individuals suffer more malaria attacks than one would expect is crucial to targeting malaria control efforts for maximum impact [2], and is also necessary for correct statistical inference [3,4]. However, the proportion of individuals in cohort studies who experience a count of zero malaria episodes is often larger than would be expected on the basis of a Poisson or negative binomial distribution, and may form a distinct sub-group, but this is less frequently considered. Zero-inflated versions of Poisson and negative binomial regression models can be used to address such situations [5], and have been used to analyse data on HIV prevention [6], sexual health [7] and cholera [8]. Use of zero-inflated methods in the study of malaria has focused mainly on spatial applications [9-11] or time series analysis [12,13] but these approaches have not been used widely to analyse prospective data from cohort studies.

Zero-inflated regression models are two-part models, comprising binary and count components [5], which explicitly model the two separate processes that may give rise to a child experiencing a count of zero malaria episodes. In the case of malaria, a child who is exposed to bites from infectious mosquitoes may not experience malaria during a particular study, because, by chance, s/he happens not to become infected or does not become unwell during the time observed. These ‘sampling’ zeroes are estimated by the count section of the model. Alternatively, a child may not experience malaria because they are never exposed to infection so cannot become unwell. These ‘certain’ zeros, estimated by the binary component of the model, are responsible for the excessive number of zero counts observed. Zero-inflated models allow these two distinct processes to be disentangled, and the fraction of the population not at risk to be estimated.

Understanding whether part of the population would remain malaria-free regardless of protective measures may be particularly important for studies of preventive interventions, such as a vaccine, when absence of an episode may be considered a success [14]. Failure to account for an unexposed fraction can lead to biased estimates of intervention effects. For interventions that may partially protect some individuals and completely protect others, differentiating partial and complete protection may be of particular interest [15-17]. This is possible within the zero-inflated model framework by including covariates in the count or binary sections of the model, respectively. Understanding what factors are associated with remaining malaria-free, particularly in areas of apparently high transmission, may be important in understanding where malaria control efforts should, and should not, be focused.

To explore these issues, data from two cohorts of Ghanaian children followed from early in infancy until two years of age were re-analysed.

## Methods

### Data

This study used data from a cluster-randomized trial of intermittent preventive treatment (IPTi) undertaken in 2,485 infants followed until two years of age in Navrongo, Ghana (described in detail in [18] and Additional file 1). Malaria transmission in Navrongo is intense and highly seasonal [19]. Data from a birth cohort in Kintampo, Ghana [20], an area of year-round high transmission [21], were used, restricting the study cohort to children followed up beyond 18 months of age (n = 733). In both studies, clinical malaria was defined as a history of fever within 48 hours (or a recorded temperature ≥37.5°C) plus parasitologically confirmed malaria infection. For this analysis, only passively detected clinical episodes were included. To avoid counting the same episode twice, malaria attacks occurring within seven days of a previous episode were discounted. To avoid making any additional assumption about the duration of post-treatment prophylaxis from the anti-malarials used for treatment, person-time at risk was not adjusted after treatment for a malaria episode.

### Statistical methods

All analyses were undertaken in Stata 12 (StataCorp, TX, USA). The count of malaria episodes per child was described first. The Kaplan-Meier method was used to estimate the proportion of children free of malaria; levelling-off of the survival curve was used as a graphical means to assess whether follow-up was sufficient to establish that children who remained malaria-free were unexposed. Several formal tests of sufficiency of follow-up have been proposed, e g, Maller and Zhou [22] and Shen [23]. The Maller and Zhou test was used to assess formal evidence of an unexposed fraction in the cohorts (Additional file 2).

Four types of model were then fitted to the data: Poisson, negative binomial (NB), zero-inflated Poisson (ZIP) and zero-inflated negative binomial (ZINB). For each model, a set of covariates were included on the basis of having a plausible association with malaria incidence. For the Navrongo trial, these were sex, intervention group (placebo or IPTi), zone of residence (urban, reference; rocky highland rural; lowland rural; irrigated rural, as defined in [19]), and season of birth (late wet season (Sep-Nov, reference); early dry season (Dec-Feb); late dry season (Mar-May); early wet season (June-Aug)). For the Kintampo study, the covariates included were as defined in [20]: sex, socio-economic group based on quintiles of asset scores (least poor as reference), rural (*vs* urban) residence, distance of residence from a health centre (≥5 km *vs* <5 km), thatched roof (*vs* non-thatched), sibling antibody titre (used as a proxy measure of exposure; based on tertiles, with low as reference) and bed net use (based on tertiles; low use as reference). Red blood cell polymorphisms were measured in a sub-group of children studied in Kintampo (Additional file 3).

In each model, person-days at risk were included in the model to account for varying exposure. Robust standard errors were used to account for the cluster-randomized design of the Navrongo trial data. The effect of assuming an inverse Gaussian distribution instead of a Gamma distribution for the heterogeneity was also explored (Additional file 4).

### Model fitting

The fitted probability distribution from each model was compared visually to the observed distribution of malaria episodes in each cohort. For the Poisson model, the deviance and Pearson goodness-of-fit tests were used to assess the null hypothesis that data were Poisson. For the NB model, a likelihood ratio test (LRT) that the overdispersion parameter, α = 0 was used formally to assess the evidence against the null hypothesis of a Poisson distribution; for the Navrongo data, this was not possible due to the use of robust standard errors, so the point estimate of α and its confidence interval were inspected.

ZIP and ZINB models were then fitted, including the same set of covariates in the count component of the model as for the Poisson and NB models. The logit component of the zero-inflated models estimates the odds of not experiencing any malaria episodes, i e, remaining malaria-free. For simplicity, only covariates that could plausibly influence whether a child never experienced malaria by two years of age were included in the logit component (for Navrongo, intervention group and zone of residence; for Kintampo, socio-economic group, rural residence, thatched roof, sibling antibody titre category and bed net use).

The Akaike information criterion (AIC) was used to compare all models. For the Kintampo data, the Vuong test was also used to assess evidence for the superiority of the zero-inflated model over its non-zero-inflated equivalent (i e, ZIP *vs* Poisson, ZINB *vs* NB), and a likelihood ratio test was used to compare the ZINB and ZIP models [5]. Having identified the most suitable model to analyse the data, the importance of the different risk factors for malaria in the two datasets were then evaluated.

## Results

### Malaria incidence

**Figure 1:**
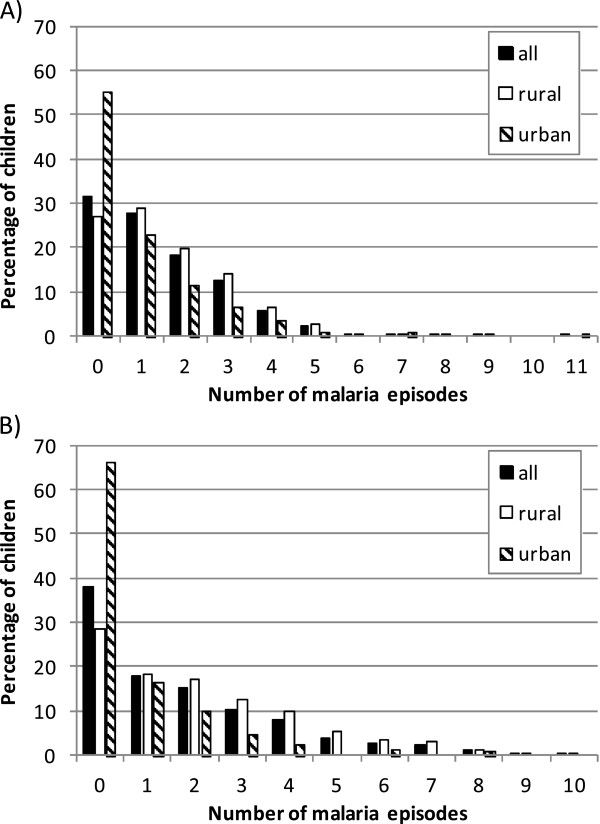
**Number of malaria attacks experienced by 24 months of age.** The figures show the number of malaria attacks experienced by 24 months of age in **A)** Navrongo and **B)** Kintampo, for all residents, and by area of residence (urban or rural).

In Navrongo, there were 3,650 malaria episodes in 4,358.2 child-years of follow-up, an incidence rate of 837.5 per 1,000 child-years (Table 1). The mean number of malaria episodes was 1.47, (range 0 to 11, variance 2.18); 31.6% of children did not experience an episode of malaria during the period of observation, whereas 22.2% experienced three or more episodes. Of children in urban areas, 55.2% remained malaria-free, compared to 27.0% of children in rural areas (Figure 1). Only 9.56% of the total burden of malaria episodes was borne by urban residents (16% of the population).

**Table 1:** Malaria incidence in the Navrongo and Kintampo infant cohorts

	**Navrongo**	**Kintampo**
Number in cohort	2,485	733
Person-years at risk	4,358.2	1,365.8
Number of malaria episodes	3,650	1,286
Malaria incidence rate	837.5	941.6
(per 1,000 person-years at risk)		
Number of malaria episodes per child	1.47, 1, (0, 11)	1.75, 1, (0, 10)
Mean, Median, (Range)		
Variance in number of malaria episodes	2.18	4.03

Children were followed from two months of age until 24 months in Navrongo, and from birth until 24 months of age in Kintampo.

In Kintampo, 1,286 episodes occurred in 1,365.8 child-years at risk, a rate of 941.6 per 1,000 child-years (Table 1). The mean number of malaria episodes was 1.75 per child (range 0 to 10, variance 4.03); 38.2% of children never experienced clinical malaria (66.1% in urban areas, 28.7% in rural areas), while 28.8% had three or more attacks. Only 9.8% of all malaria episodes occurred among urban residents (25.4% of the population).

**Figure 2:**
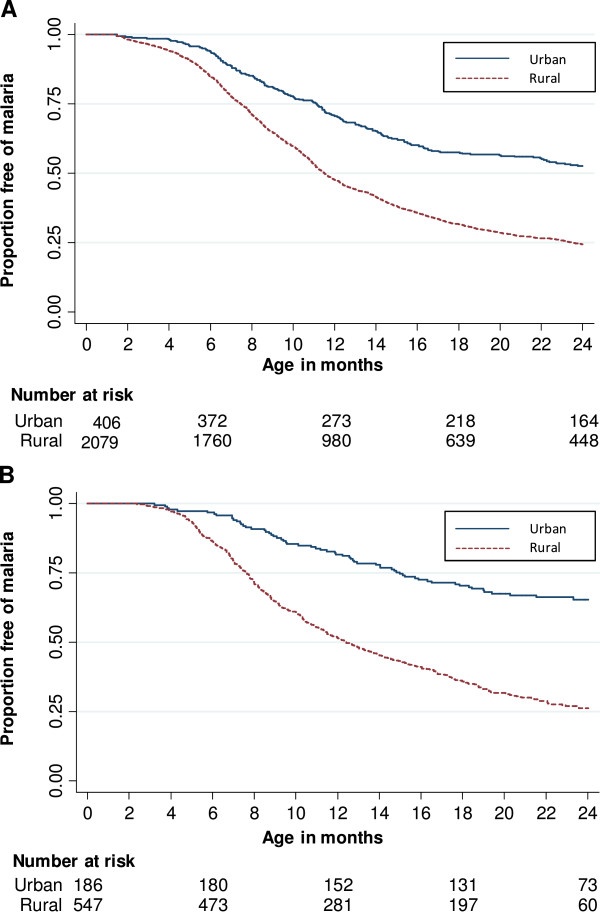
**Time to first malaria episode according to place of residence.** Figures show Kaplan-Meier estimate of time to first malaria episode in urban and rural areas for **A)** Navrongo and **B)** Kintampo cohorts. Tables show number of children remaining at risk at 6-month intervals. For clarity of presentation, the three rural areas in Navrongo (rocky highland, lowland rural, irrigated rural) were combined. Malaria incidence rates on the same time scale are shown in the Additional files.

Analysis of time to first event using the Kaplan-Meier method indicated that a sub-group of children was apparently at no risk of malaria in both study sites; this sub-group was much larger in urban areas (Figure 2). The levelling off of the survival curves was not due to changes in transmission in the study areas over time, as indicated by the continued high incidence overall in the second year of life (see Additional file 1). The Maller and Zhou non-parametric test provided strong evidence against the null hypothesis that the whole population is susceptible (i e, there was evidence of an unexposed fraction, see Additional file 2). However, the second part of the test (which assesses whether there is sufficient follow-up time to reliably establish the existence of an unexposed sub-group) was usually indeterminate, except for urban residents in Navrongo, where there was evidence of sufficient follow-up (Additional file 2).

### Comparison of different regression models

**Figure 3:**
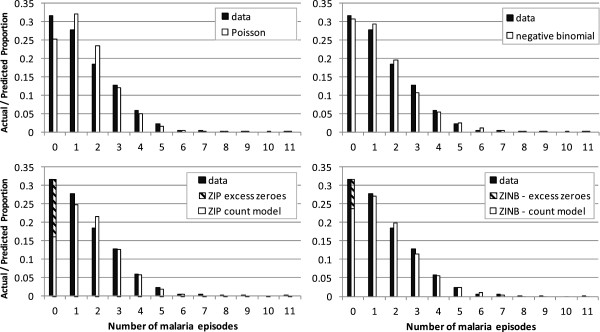
Poisson, negative binomial, ZIP and ZINB model fits to data - Navrongo.

**Figure 4:**
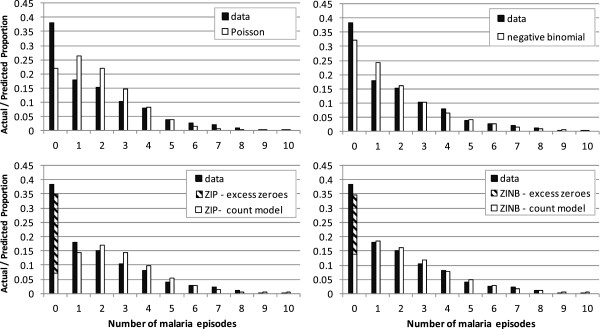
Poisson, negative binomial and ZINB model fits to data – Kintampo.

In both cohorts, the Poisson and negative binomial models tended to underestimate the number of children with zero malaria attacks, and overestimate the number with one malaria attack (Figures 3 and 4). This was most marked in the Kintampo data. The ZIP model estimated the proportion of zero counts better, but tended to underestimate the proportion of children with a single malaria attack, and overestimate the number with two attacks. The ZINB model provided the closest fit to the data in both cohorts.

For the Navrongo data, there was strong evidence against the null hypothesis that the data was Poisson (both deviance and Pearson goodness-of-fit P < 0.0001), with overdispersion parameter, α = 0.25 (95% CI: 0.19, 0.34). The ZINB model had the lowest value of the AIC (Table 2), with the next lowest being the NB model (difference in AIC 22.8), providing very strong grounds for preferring the ZINB model [24]. Accounting for excess zeroes was particularly important among urban residents, where 33.5% of children were estimated to be at zero risk, compared to 2.97% of rural residents (7.96% overall).

**Table 2:** Log-likelihoods and Information criteria for the regression models

	**Log-likelihood (null)**	**Log-likelihood (model)**	**Model degrees of freedom**	**AIC**
**Navrongo**				
Poisson	−4072.6	−3982.8	9	7983.6
Negative binomial (NB)	−3983.3	−3918.1	10	7856.2
Zero-inflated poisson (ZIP)	−3934.0	−3917.9	14	7863.8
Zero-inflated negative binomial (ZINB)	−3916.8	−3901.7	15	7833.3
**Kintampo**				
Poisson	−1432.2	−1291.7	13	2609.3
Negative binomial (NB)	−1284.0	−1213.2	14	2454.3
Zero-inflated poisson (ZIP)	−1238.7	−1212.3	24	2472.6
Zero-inflated negative binomial (ZINB)	−1215.8	−1197.5	25	2444.9

AIC = Akaike information criterion.

For the Kintampo data, there was strong evidence against the null hypothesis that the data was Poisson (both deviance and Pearson goodness-of-fit P < 0.0001), with α = 0.43 (95% CI: 0.32, 0.57), LRT p < 0.001. The ZINB model provided the best fit to the data, with the smallest AIC (Table 2). The NB model provided the next best fit (difference in AIC 8.5), again providing strong grounds for preferring the ZINB model [24]. Of urban residents, 46.6% were estimated to be at no risk, compared to 12.8% of rural residents (21.1% overall). The Vuong test, comparing the ZINB and NB models, indicated that the ZINB model gave a superior fit to the NB model, p = 0.0017, and the LRT comparing ZINB and ZIP indicated the ZINB model to be superior, p < 0.0001. The ZINB model was therefore used for subsequent stages of the analysis of both datasets.

### Interpretation of ZINB regression model output

#### Navrongo

IPTi reduced malaria incidence (IRR 0.87 (95% CI: 0.78, 0.97); p = 0.01), but was not associated with odds of never experiencing malaria, although the CI for the OR was wide (OR 1.16 (0.46, 2.89); p = 0.755) (Table 3). Residence in the lowland or irrigated rural areas was associated with an increased incidence of malaria compared to the urban area (IRR 1.27 (1.08, 1.51), p = 0.005 and 1.27 (1.05, 1.54), p = 0.016, respectively). A similar point estimate was obtained for residence in the rocky highland rural area, although the CI overlapped unity (IRR 1.22 (0.95, 1.58), p = 0.123). Residence in the lowland rural and irrigated rural area also reduced the odds of never experiencing malaria by 24 months of age (OR 0.04 (0, 0.97), p = 0.048 and 0.08 (0.01, 0.85), p = 0.036, respectively). Malaria incidence was similar by season of birth, except for children born late in the dry season (Mar-May), who experienced a lower incidence of malaria (IRR 0.86 (0.77, 0.97), p = 0.015, compared to children born in the late wet season. Gender was not strongly associated with either incidence rate of malaria or the odds of remaining free of malaria.

**Table 3:** Zero-inflated negative binomial regression output for the Navrongo cohort

**Negative binomial component**			**Logistic component**		
**Incidence rate ratios (IRR)**			**Odds ratio (OR) for not having malaria**		
	**IRR (95% CI)**	**P**		**OR (95% CI)**	**p**
**IPTi**	0.87 (0.78, 0.97)	0.01	**IPTi**	1.16 (0.46, 2.89)	0.755
**Zone of residence**			**Zone of residence**		
urban	-		urban	-	
rocky highland	1.22 (0.95, 1.58)	0.123	rocky highland	0.22 (0.02, 2.85)	0.247
lowland rural	1.27 (1.08, 1.51)	0.005	lowland rural	0.04 (0, 0.97)	0.048
irrigated rural	1.27 (1.05, 1.54)	0.016	irrigated rural	0.08 (0.01, 0.85)	0.036
**Season of birth**			-		
late wet	-				
early dry	0.99 (0.88, 1.10)	0.8			
late dry	0.86 (0.77, 0.97)	0.015			
early wet	0.94 (0.84, 1.06)	0.305			
**Sex** (female *vs* male)	0.96 (0.90, 1.04)	0.335	-		

Table shows output from the zero-inflated negative binomial regression model. Incidence rate ratios are from the count component and odds ratios are from the logistic component.

CI = confidence interval; IPTi = intermittent preventive treatment in infants.

#### Kintampo

Rural residence was strongly associated with an increased incidence rate of malaria (IRR 1.58 (1.18, 2.13), p = 0.002) and also with reduced odds of never experiencing malaria by 24 months of age (OR 0.23 (0.1, 0.55), p = 0.001, Table 4). Socio-economic status influenced the rate of malaria attacks, with strong evidence that the three lowest quintiles all experienced higher malaria incidence. Fitting SES as a linear trend suggested an increase in incidence for each unit decrease in SES group (IRR 1.08 (1.01, 1.15), p = 0.02), and a reduced odds of never experiencing malaria (OR 0.59 (0.42, 0.85), p = 0.004). Other factors including sex, distance from health centre, roof construction, sibling antibody response category and bed net usage were not associated with malaria incidence rate, nor with odds of not experiencing malaria.

**Table 4:** Zero-inflated negative binomial regression output for the Kintampo data

**Negative binomial component**			**Logistic component**		
**Incidence rate ratios (IRR)**			**Odds ratio (OR) for not having malaria**		
	**IRR (95% CI)**	**p**		**OR (95% CI)**	**p**
**Rural residence**	1.64 (1.21, 2.20)	0.001	**Rural residence**	0.25 (0.10, 0.58)	0.001
**Sex** (female vs. male)	0.92 (0.79, 1.07)	0.259	**-**		
**Distance from** **health centre**					
(≥5 km vs. < 5 km)	0.92 (0.78, 1.08)	0.321	-		
**Thatched roof**	1.11 (0.93, 1.32)	0.25	**Thatched roof**	1.27 (0.51, 3.16)	0.612
**SES**			**SES**		
Least poor	-		Least poor	-	
Less poor	1.51 (1.01, 2.24)	0.044	Less poor	0.59 (0.23, 1.53)	0.276
Poor	1.71 (1.18, 2.49)	0.005	Poor	0.38 (0.14, 1.05)	0.063
More poor	1.68 (1.15, 2.46)	0.008	More poor	0.34 (0.11, 1.05)	0.062
Most poor	1.65 (1.14, 2.41)	0.009	Most poor	0.07 (0, 1.67)	0.101
**Sibling antibody response category**			**Sibling antibody response category**		
Low			Low		
Medium	1.03 (0.84, 1.26)	0.77	Medium	1.28 (0.57, 2.87)	0.549
High	1.13 (0.92, 1.38)	0.241	High	1.02 (0.43, 2.44)	0.964
**Bed net use**			**Bed net use**		
Low			Low		
Medium	1.07 (0.87, 1.32)	0.526	Medium	0.86 (0.37, 1.98)	0.723
High	1.17 (0.95, 1.45)	0.138	High	0.54 (0.19, 1.52)	0.244

Table shows output from the zero-inflated negative binomial regression model. Incidence rate ratios are from the count component and odds ratios are from the logistic component.

CI = confidence interval; SES = socio-economic status.

## Discussion

Including a zero-inflation component improved the fit of negative binomial models and allowed more meaningful interpretation of the association of malaria with different risk factors. ZINB models have not been used widely in malaria cohort studies, despite the fact that their formulation allows for two well-accepted aspects of malaria epidemiology: overdispersion (a greater degree of variability between individuals than would be expected on the basis of a given statistical model) and zero-inflation (a larger number of children remaining free of malaria than would be expected if all children are genuinely at risk). However, given that these models can be fitted easily in standard statistical packages, this approach could be used more widely to disentangle the different ways that risk factors influence a child’s chances of developing malaria.

In both of the study cohorts, residence in a rural area was a clear risk factor for higher malaria incidence rates, consistent with other studies [25,26]. Urban residents were at substantially higher odds of never experiencing malaria. The relatively large fraction of children who did not experience malaria in both cohorts suggests that a considerable proportion of children, predominantly urban residents, are at no malaria risk, despite the fact that these studies took place in areas of Ghana with very high malaria transmission [19,21]. This adds to a growing body of evidence that malaria can be focal in areas of high transmission [26] in addition to areas of lower endemicity [2,27]. In Kintampo, higher socio-economic status was associated with lower incidence rates, and there was evidence of decreasing odds of remaining malaria-free with lower SES when this was fitted as a single linear term. Given the well-known links between urban/rural residence and relative wealth, it is likely that these two factors are inter-related.

IPTi reduces the incidence rate of malaria. There was no evidence from these analyses that some children were completely protected, and although the CI was wide, this fits with the rationale of IPTi as periodic chemoprevention that allows infection (and development of immunity) between courses [28]. Identification and separation of the influence of factors that provide partial and complete protection is of major interest for the analysis of the results of malaria vaccine trials, since this could help understand the mechanism by which a particular vaccine provides protection [4].

The lower incidence of malaria among children born late in the dry season in Navrongo could be due to protection from maternal immunity and foetal haemoglobin which lasts until around six months of age [29,30], a similar length of time as the rainy season. This effect would balance over the course of childhood, but not during the course of a cohort followed up to a fixed age. This idea is supported by the finding that month and season of birth were not associated with malaria incidence in Kintampo, where malaria transmission is perennial (data not shown).

An analogous, analytical approach to zero-inflated models is the use of cure or mixture survival analysis models [22], which also assume that a proportion of the population is not susceptible to the outcome of interest. Halloran *et al.* developed frailty mixing models, limited to survival analysis of first episodes [16]. This was extended recently by Xu *et al.* to multiple episodes [31]. These approaches have the potential advantage over zero-inflated models that they can allow for event dependence and variation in the hazard with time. In the study of Xu *et al.*, which also used the Navrongo data, IPTi was found to provide complete protection to some children, as well as the partial protection seen in this study. It may be that the information provided by timing of events gives greater power to identify factors enabling complete protection.

The Akaike information criterion (AIC) is used as a guide to comparing models. The large differences in AIC to the next best fitting model (the negative binomial) provide very strong grounds for preferring the ZINB model [24]. The advantages of the zero-inflated model were retained when heterogeneity between individuals was modelled as inverse-Gaussian rather than a gamma distribution, suggesting that the excess zeroes cannot be accounted for by simply assuming a different distribution of heterogeneity between individuals (Additional file 4).

## Conclusion

Zero-inflated models can help understand the mechanism by which different risk factors influence malaria, either by preventing or allowing exposure, influencing the level of exposure, or both. The protective effect of urban residence on malaria incidence was partly due to decreasing incidence rates in children who were exposed, and partly because living in an urban area prevents some children from being exposed at all. This finding is an elaboration of what would have been found using only a negative binomial regression model, i e, that urban residence decreases malaria incidence. Other studies to investigate malaria incidence, or other diseases with similar biology, could employ these models to better understand how risk factors affect clinical outcomes. Given the known features of malaria epidemiology, the use of zero-inflated models should be considered more widely than they are at present.

These findings are consistent with existing knowledge and emphasize the importance of targeted malaria control. Delivery strategies that reach only easily accessed urban populations will have less impact than if targeted successfully at rural areas. Furthermore, these results show that protecting some urban residents may have no impact at all on the overall malaria burden, because some urban residents are essentially at no risk even if not protected. These results therefore have implications for malaria burden estimates, and underline the importance of delivery strategies that reach the most disadvantaged, and achieve high coverage in rural areas.

## Abbreviations

AIC: Akaike information criterion; IPTi: Intermittent preventive treatment in infants; IRR: Incidence rate ratio; LRT: Likelihood ratio test; NB: Negative binomial; OR: Odds ratio; SES: Socio-economic status; ZINB: Zero-inflated negative binomial; ZIP: Zero-inflated poisson.

## Competing interests

The authors declare that they have no competing interests.

## Authors’ contributions

MEC conceived and designed the study, analysed the data, and wrote the first draft of the manuscript; KPA supervised field activities for the Kintampo cohort study and helped analyse the trial data. SOA and DC supervised field activities in the Navrongo study and assisted with interpretation of the trial data. BMG contributed to study design and writing of the draft manuscript. PJM contributed to study design, analysis of the data and writing of the draft manuscript. All authors contributed to interpretation of the analyses and revised the draft manuscript.

## Supplementary Material

Additional file 1 **Additional Details of Cohort Studies.** Description: Additional details of the cohort studies analysed in the manuscripts, and malaria incidence rates over the period of the studies. Click here for file

Additional file 2 **Tests for presence of immunes & sufficient follow-up.** Description: Results of non-parametric method developed by Maller and Zhou to assess for presence of immunes and sufficiency of follow-up. Click here for file

Additional file 3 Effect of red blood cell polymorphisms on malaria incidence Description: Results of analyses investigating association of red blood cell polymorphisms and malaria incidence. Click here for file

Additional file 4 **Alternative Distribution for Heterogeneity.** Description: Comparison of results from zero-inflated inverse Gaussian and zero-inflated negative binomial models. Click here for file
