# Supplementary material for: Analysis of partial and complete protection in malaria cohort studies
Source: Malar J. 2013 Oct 5;12:355. doi: 10.1186/1475-2875-12-355 (PMC3850882; doi:10.1186/1475-2875-12-355)
Supplement: Additional file 1 — Additional Details of Cohort Studies. Description: Additional details of the cohort studies analysed in the manuscripts, and malaria incidence rates over the period of the studies. [file 1475-2875-12-355-S1.doc]

**Additional File 1: Additional Details of Cohort Studies**

**Navrongo**

A cluster-randomised trial of intermittent preventive treatment (IPTi) in 2485 infants was undertaken in Navrongo, Ghana, between 2000-2004 [1]. Children were enrolled at two months of age. Four doses of sulfadoxine-pyrimethamine or placebo were given to infants at healthcare contacts at 3, 4, 9 and 12 months of age. Infants were followed up until two years of age. Malaria transmission in Navrongo is intense, with a reported EIR of 418 at the time of study, and a seasonal peak between June and November [2]. Treatment for malaria at the time of the study was predominantly chloroquine, with quinine used to manage severe malaria.

**Kintampo**

Data from a birth cohort completed in Kintampo, Ghana were also used [3]. This study site has a very high burden of malaria and year-round malaria transmission [4]. For this study, only clinical episodes that were reported passively at study clinics were analyzed. Because some children left the cohort after one year, only children followed-up beyond 18 months of age were included in the analysis (n=733). Treatment for malaria in study children was amodiaquine-artesunate or artemether-lumefantrine, following national guidelines.

**Malaria incidence rate over the study period**

In both Navrongo and Kintampo, incidence of malaria was relatively low in the first few months of life, increasing steeply from 3-6 months of age and remaining high thereafter (figure S1). Overall incidence of malaria remained high in the second year of life. Flattening of the Kaplan-Meier plots (figure 2, main paper) is, therefore, not due to changes in transmission in the study areas over time.

**Figure S1. Malaria incidence rate (all episodes) during the study period**

**Navrongo**

**Kintampo**

**References**

1. Chandramohan D, Owusu-Agyei S, Carneiro I, Awine T, Amponsa-Achiano K, Mensah N, Jaffar S, Baiden R, Hodgson A, Binka F, Greenwood B: **Cluster randomised trial of intermittent preventive treatment for malaria in infants in area of high, seasonal transmission in Ghana.** *British Medical Journal* 2005, **331:**727-733.

2. Appawu M, Owusu-Agyei S, Dadzie S, Asoala V, Anto F, Koram K, Rogers W, Nkrumah F, Hoffman SL, Fryauff DJ: **Malaria transmission dynamics at a site in northern Ghana proposed for testing malaria vaccines.** *Trop Med Int Health* 2004, **9:**164-170.

3. Asante KP, Owusu-Agyei S, Cairns ME, Dodoo D, Boamah E, Gyasi R, Adjei G, Gyan B, Agyeman-Budu A, Dodoo T, Mahama E, Amoako N, Dosoo DK, Koram K, Greenwood B, Chandramohan D: **Placental malaria and the risk of malaria in infants in a high malaria transmission area in Ghana: a prospective cohort study.** *J Infectious Dis* 2013, **in press**.

4. Owusu-Agyei S, Asante KP, Adjuik M, Adjei G, Awini E, Adams M, Newton S, Dosoo D, Dery D, Agyeman-Budu A, Gyapong J, Greenwood B, Chandramohan D: **Epidemiology of malaria in the forest-savanna transitional zone of Ghana.** *Malar J* 2009, **8:**220.
