# Supplementary material for: Analysis of partial and complete protection in malaria cohort studies
Source: Malar J. 2013 Oct 5;12:355. doi: 10.1186/1475-2875-12-355 (PMC3850882; doi:10.1186/1475-2875-12-355)
Supplement: Additional file 2 — Tests for presence of immunes & sufficient follow-up. Description: Results of non-parametric method developed by Maller and Zhou to assess for presence of immunes and sufficiency of follow-up. [file 1475-2875-12-355-S2.doc]

**Additional file 2:** **Non-parametric tests for presence of immunes, and sufficient period of follow-up**

Table S1 shows results from the non-parametric method for from Maller and Zhou [1] to test for the presence of an immune fraction in the population, and to test for sufficient follow-up to make a firm conclusion regarding the presence or absence of immunes. The test comprises two parts, with both results needed for full interpretation.

The first test assesses evidence against the null hypothesis that there are no ‘immunes’ in the population (in the context of this study, this can be interpreted as the null hypothesis that there are no non-susceptible children in the cohort, i.e. all children would get malaria if follow-up was sufficient).

The second stage tests the null hypothesis of sufficient follow up, and the complementary null hypothesis of insufficient follow-up. If neither null hypothesis is rejected at the second stage, the results regarding sufficiency of follow-up are considered indeterminate.

In brief, the test proceeds as follows, using urban residents in the Navrongo cohort as an example.

**Part 1: Test for presence of immunes (Hypothesis H_01_)**

1. Let p­_n_ be the Kaplan-Meier estimate of the probability of failure at the end of follow-up. For urban residents in Navrongo, p_n_ = 0.473

2. The proportion of individuals who are censored (those who exit follow-up without experiencing the event of interest) is c_n_ = 1-p­_n_ . For this example, c_n_ = 1 – 0.473 = 0.527 (i.e. 52.7% of urban residents were censored without experiencing malaria).

3. Calculate the mean of the censoring distribution from c_n_ = φ / (φ + μ), where φ = survival mean and μ = censoring mean. Assuming a survival mean of 1, c_n_ = 1 /(1+μ), and μ = (1/ c_n_) – 1.

For example, assuming exponential censoring if c_n_ = 0.527, φ = 1, μ = (1/ 0.527) – 1 = 0.90 ≈ 1

To assume uniform censoring work out the mean as above, then use the uniform censoring distribution with the same mean noting that Uniform [0,B] has mean B/2. For this example μ = 0.9 ≈ 1 and therefore B = 2.

4. Look up in table A1 or A2 in Maller and Zhou [1] the percentage point which corresponds to the closest value of n to the sample size, and with the corresponding censoring mean. To assume exponential censoring, the censoring mean μ is used to look up a value in Table A1. To assume uniform censoring use B and table A2.

For n, looking up the value below the observed n is conservative.

For censoring, using a lower estimate of μ or B is conservative (since this implies heavier censoring, and expected p_n_ is therefore lower)

5. The 1%, 5%, 10% and 20% critical value of p_n_ is shown in the table. If the observed value of p_n_ (estimated susceptible proportion) is smaller than the percentage point in the table then H_01_ is rejected and one can conclude that immunes are present. Otherwise, H_01_ is accepted i.e. that no immunes are present.

In this example, the 1% critical value of p_n_ was 0.8725 (for exponential censoring) and 0.931 (for uniform censoring). The Kaplan-Meier estimate of p­_n_, 0.473, is clearly lower than either critical value, providing strong evidence that immunes are present.

**Part 2: Test for sufficient follow-up (Hypothesis H_02_)**

This approach uses the amount of time after the last event until the end of the study, and determines if this is long enough to establish whether or not there will be any more events.

1. If T is the date of last follow-up (the end of the study), and t_max_ is the date of the last event, the length of the interval at the end of the study during which no events were observed is D = T - t_max_.

Next the time prior to t_max_ is considered. Count back a further D days, denoting the earlier time T_0_ .

T0 t_max_ T

<------ D ------><------ D ------>

[ events counted ]

The test statistic, denoted q_n_, is the proportion of study subjects who had a first event in the interval T_0_ to t_max_, including events on the boundaries of the interval. In this example, the last event among the 406 urban residents occurred at 1.981 years of age (7 days before reaching 2 years of age). Two children had their first event in the time interval between T_0_ and t_max_ (the one at tmax and one other between T_0_ and t_max_), thus q_n_=2/406 = 0.00246.

Using q_n_ and p_n_ (the maximum of the Kaplan Meier failure function, from part 1), and the sample size, n, use the tables tables B1 to B8, to get P-values. A q_n_ value above that for the 95th percentile indicates evidence to reject the null of insufficient follow-up (i.e. indicating that there is sufficient follow-up to establish that there will be no more events). A q_n_ value below the 5^th^ percentile indicates evidences to reject the complementary null hypothesis of sufficient follow-up (i.e. follow-up is insufficient). A value of q_n_ between the 10^th^ and 90^th^ percentile give no clear evidence either way. In this example, the value 0.00246 is below the 5% critical value for q_n_ , suggesting that follow-up is sufficient.

Taken together, the conclusion is therefore that follow-up is sufficient to determine whether or not there is an immune fraction (part 2), and that there is a non-susceptible fraction (part 1).

**Results**

In both Navrongo and Kintampo, the first stage of the test indicated strong evidence against the null hypothesis that there are not any non-susceptible children in the cohort. This is because the final Kaplan-Meier failure estimate, p_n_ is well below the critical value of p_n_ from the tables in Maller and Zhou.

In Navrongo, the test for sufficient follow-up gave conclusive results for urban residents only, where follow-up was sufficient and thus the conclusion is that there is an unexposed fraction in the population. In rural areas and in Navrongo overall, there was a malaria case on the last day of follow-up, which prevents the second stage being used. Ignoring the failure that occurred on the last day of follow-up, there was also evidence of sufficient follow-up, but this should be interpreted with caution.

In Kintampo, all results for the second stage were indeterminate, i.e. it is not possible to formally conclude whether follow-up is sufficient or insufficient.

Table S1. Non-parametric test for presence of immunes and a sufficient period of follow-up

|  | **Navrongo** | | | **Kintampo** | | |
| --- | --- | --- | --- | --- | --- | --- |
|  | **All children** | **Rural** | **Urban** | **All children** | **Rural** | **Urban** |
| **1. Presence of immunes** |  |  |  |  |  |  |
| Number of children | 2485 | 2079 | 406 | 733 | 547 | 186 |
| p_n_ **(KM estimate of failure probability at end of study)** | **0.710** | **0.756** | **0.473** | **0.635** | **0.737** | **0.346** |
| Proportion censored (c_n_) | 0.290 | 0.244 | 0.527 | 0.365 | 0.263 | 0.654 |
| μ = (1/ c_n_ ) – 1 | 2.45 | 3.09 | 0.90 | 1.74 | 2.80 | 0.53 |
|  |  |  |  |  |  |  |
| μ, exponential censoring | 2 | 3 | 1 | 2 | 3 | 1 |
| percentage point | 1% | 1% | 1% | 1% | 1% | 1% |
| **critical value of** p_n_ | **0.9746** | **0.9866** | **0.8725** | **0.9601** | **0.9781** | **0.816** |
|  |  |  |  |  |  |  |
| Β, uniform censoring | 4 | 6 | 2 | 4 | 6 | 2 |
| percentage point A2 | 1% | 1% | 1% | 1% | 1% | 1% |
| **critical value of** p_n_ | **0.971** | **0.982** | **0.931** | **0.961** | **0.974** | **0.905** |
|  |  |  |  |  |  |  |
| **Interpretation** | In all cases, strong evidence against the null hypothesis of no immunes | | | | | |
|  |  |  |  |  |  |  |
| **2. Sufficient or**  **insufficient follow-up** | **All children** | **Rural** | **Urban** | **All children** | **Rural** | **Urban** |
| T (time follow up ended) | 2 | 2 | 2 | 2 | 2 | 2 |
| tmax (time of last event) | 1.995* | 1.995* | 1.981 | 1.963 | 1.963 | 1.941 |
| D: interval T-tmax (years) | 0.005 | 0.005 | 0.019 | 0.037 | 0.037 | 0.059 |
| interval in days | 2 | 2 | 7 | 14 | 14 | 22 |
| tmax-D (years) | 1.99 | 1.99 | 1.962 | 1.926 | 1.926 | 1.882 |
| n events in interval tmax-D | 1 | 1 | 1 | 3 | 2 | 1 |
| **q_n_** | **0.00040** | **0.00048** | **0.00246** | **0.00409** | **0.00366** | **0.00538** |
|  |  |  |  |  |  |  |
| percentage point | 95% | 95% | 95% | 95% | 95% | 95% |
| **critical value of q_n_ to reject hypothesis of sufficient follow-up** | **0.023** | **0.063** | **0.04** | **0.0386** | **0.178** | **0.105** |
|  |  |  |  |  |  |  |
| percentage point | 5% | 5% | 5% | 5% | 5% | 5% |
| **critical value of q_n_ to reject null of**  **insufficient follow-up** | **0.001** | **0.001** | **0.0025** | **0.0014** | **0.002** | **0.005** |
|  |  |  |  |  |  |  |
| **Interpretation** | * q_n is below 5% critical value – reject null of insufficient follow-up, i.e. **conclude follow up is sufficient** | | q_n is just below 5% critical value - **follow up is sufficient** | Interpretation: q_n is between the 5% and 95% critical values, so **cannot reject either null hypothesis regarding length of follow-up**. | | |

Abbreviations: KM, Kaplan-Meier. * 1 failure occurred on the last day of follow-up, i.e. tmax=T. When this occurs, this method cannot be used. Results shown are for the previous failure, but should be interpreted with caution.

**References**

1. Maller R, Zhou X: *Survival analysis with long-term survivors.* New York: Wiley; 1996.
