# Supplementary material for: Analysis of partial and complete protection in malaria cohort studies
Source: Malar J. 2013 Oct 5;12:355. doi: 10.1186/1475-2875-12-355 (PMC3850882; doi:10.1186/1475-2875-12-355)
Supplement: Additional file 3 — Effect of red blood cell polymorphisms on malaria incidence Description: Results of analyses investigating association of red blood cell polymorphisms and malaria incidence. [file 1475-2875-12-355-S3.doc]

**Additional File 3. Effect of red blood cell polymorphisms on malaria incidence**

Certain red blood cell polymorphisms, such as the possession of sickle cell trait, and glucose-6-phosphate dehydrogenase deficiency, provide strong protection against severe malaria. These factors may influence incidence of clinical malaria, although the protection provided by these genetic traits is usually found to be less than that provided against severe malaria. [1].

These genetic factors were measured in a sub-group of children enrolled in the Kintampo cohort. Haemoglobin S and Glucose-6-phosphate dehydrogenase (G6PD) deficiency were assessed by genotyping, and categorised into phenotype groups using standard methods [2-6]. Haemoglobin S status was categorised as normal (genotype AA), sickle cell trait (carrier, for genotypes AS and AC), and sickle cell disease (for genotypes SS or CC). G6PD was defined as normal (genotypes AB or BB), mild deficiency (for genotypes AA, A-B, and AA-) or severe deficiency (for genotypes A-A-).

The percentage of children who did not experience malaria was similar among those with normal Hb, children with sickle cell trait, and sickle cell disease (figure S2). The distribution of malaria episodes was also similar in children with normal glucose-6-phosphate dehydrogenase, and those with mild and severe deficiency (figure S3). These two variables were not associated with either incidence rate of malaria infection, or odds of remaining malaria free, in either univariate or multivariate analysis.

**Figure S2. Distribution of number of malaria episodes by Haemoglobin S status**

Number by category: Normal = 320, Sickle Cell Trait = 77, Sickle Cell Disease = 36, Total = 433.

**Figure S3. Distribution of number of malaria episodes by G6PD deficiency status**

Number by category: Normal = 272, Mild deficiency = 162, Severe deficiency = 48, Total = 482.

**References**

1. Billo MA, Johnson ES, Doumbia SO, Poudiougou B, Sagara I, Diawara SI, Diakite M, Diallo M, Doumbo OK, Tounkara A, Rice J, James MA, Krogstad DJ: Sickle cell trait protects against Plasmodium falciparum infection. *Am J Epidemiol* 2012, 176 Suppl 7:S175-185.

2. Danquah I, Ziniel P, Eggelte TA, Ehrhardt S, Mockenhaupt FP: Influence of haemoglobins S and C on predominantly asymptomatic Plasmodium infections in northern Ghana. *Trans R Soc Trop Med Hyg* 2010, 104:713-719.

3. Frank JE: Diagnosis and management of G6PD deficiency. *Am Fam Physician* 2005, 72:1277-1282.

4. Meissner PE, Coulibaly B, Mandi G, Mansmann U, Witte S, Schiek W, Muller O, Schirmer RH, Mockenhaupt FP, Bienzle U: Diagnosis of red cell G6PD deficiency in rural Burkina Faso: comparison of a rapid fluorescent enzyme test on filter paper with polymerase chain reaction based genotyping. *Br J Haematol* 2005, 131:395-399.

5. Clark TG, Fry AE, Auburn S, Campino S, Diakite M, Green A, Richardson A, Teo YY, Small K, Wilson J, Jallow M, Sisay-Joof F, Pinder M, Sabeti P, Kwiatkowski DP, Rockett KA: Allelic heterogeneity of G6PD deficiency in West Africa and severe malaria susceptibility. *Eur J Hum Genet* 2009, 17:1080-1085.

6. Carter N, Pamba A, Duparc S, Waitumbi JN: Frequency of glucose-6-phosphate dehydrogenase deficiency in malaria patients from six African countries enrolled in two randomized anti-malarial clinical trials. *Malar J* 2011, 10:241.
