# Supplementary material for: Analysis of partial and complete protection in malaria cohort studies
Source: Malar J. 2013 Oct 5;12:355. doi: 10.1186/1475-2875-12-355 (PMC3850882; doi:10.1186/1475-2875-12-355)
Supplement: Additional file 4 — Alternative Distribution for Heterogeneity. Description: Comparison of results from zero-inflated inverse Gaussian and zero-inflated negative binomial models. [file 1475-2875-12-355-S4.doc]

**Additional File 4. Alternative distribution for heterogeneity**

Part of the explanation for the apparent superiority of the ZINB model over the NB model may be because the gamma distribution is not an adequate description of the heterogeneity between individuals. The effect of assuming an inverse Gaussian distribution for the heterogeneity was therefore explored, i.e. fitting Poisson inverse-Gaussian (PIG) and Zero-inflated Poisson inverse-Gaussian (ZIPIG) models, using the user-written stata commands pigreg and zipig, [1], (available at <http://works.bepress.com/joseph_hilbe/subject_areas.html>).

Assuming an inverse-Gaussian frailty rather than a gamma frailty did not make any important changes to the parameter estimates (table S2 and S3). In both datasets, the AIC was similar between the ZINB and ZIPIG models; indicating no clear preference between these different models, both of which account for zero-inflation and heterogeneity. The PIG model in general is appropriate when modelling correlated count data which are very highly right skewed [2], so could be considered in certain situations. However, a practical advantage of the ZINB model over the ZIPIG model is that the ZINB model is more widely available in standard software packages [2]. Commands to implement ZINB in Stata allows adjustment for clustering which was not possible with the ZIPIG model.

**Table S2. Comparison of zero-inflated negative binomial and zero-inflated Poisson inverse-Gaussian models – Navrongo**

|  | **ZINB** | | **ZIPIG** | |
| --- | --- | --- | --- | --- |
| **Count component** | **IRR** | **p-value** | **IRR** | **p-value** |
| Intervention | 0.87 (0.80, 0.94) | 0.001 | 0.87 (0.80, 0.94) | 0.001 |
|  |  |  |  |  |
| **Zone of residence** |  |  |  |  |
| urban |  |  |  |  |
| rocky highland | 1.22 (0.91, 1.65) | 0.179 | 1.23 (0.91, 1.65) | 0.175 |
| lowland rural | 1.27 (1.07, 1.51) | 0.006 | 1.28 (1.08, 1.52) | 0.005 |
| irrigated rural | 1.27 (1.06, 1.52) | 0.01 | 1.27 (1.06, 1.53) | 0.009 |
|  |  |  |  |  |
| **Season of birth** |  |  |  |  |
| late wet |  |  |  |  |
| early dry | 0.99 (0.89, 1.09) | 0.774 | 0.99 (0.89, 1.09) | 0.793 |
| late dry | 0.86 (0.78, 0.96) | 0.007 | 0.86 (0.78, 0.96) | 0.007 |
| early wet | 0.94 (0.84, 1.05) | 0.287 | 0.94 (0.85, 1.05) | 0.305 |
|  |  |  |  |  |
| Sex (female vs. male) | 0.96 (0.89, 1.04) | 0.348 | 0.97 (0.89, 1.04) | 0.36 |
|  |  |  |  |  |
| **Binary component** | **OR** | **p-value** | **OR** | **p-value** |
| Intervention | 1.16 (0.59, 2.28) | 0.674 | 1.16 (0.61, 2.21) | 0.656 |
|  |  |  |  |  |
| **Zone of residence** |  |  |  |  |
| urban |  |  |  |  |
| rocky highland | 0.22 (0.04, 1.24) | 0.086 | 0.24 (0.05, 1.14) | 0.072 |
| lowland rural | 0.04 (0.00, 0.63) | 0.022 | 0.06 (0.01, 0.30) | 0.001 |
| irrigated rural | 0.08 (0.01, 0.49) | 0.007 | 0.10 (0.02, 0.38) | 0.001 |

AIC for ZINB model 7833.3, for ZIPIG model 7832.6; AIC for standard Poisson Inverse Gaussian (not shown) 7857.7. Vuong test of ZINB vs. standard negative binomial: z = 2.73 P = 0.0031, Vuong test of ZIPIG vs. Poisson inverse gaussian: z = -13.01 P = 1.0

Note that the user-written zero-inflated Poisson inverse Gaussian model does not allow use of robust standard errors to allow for the cluster-randomised design of the Navrongo study, so both sets of estimates presented in this comparison come from models that do not account for clustering. By comparison with the ZINB model which does allow for clustering (table 3), allowing for clustering does not appear to make important differences to the estimates.

**Table S3. Comparison of zero-inflated negative binomial and zero-inflated Poisson inverse-Gaussian models – Kintampo**

| **Kintampo** | **ZINB** | | **ZIPIG** | |
| --- | --- | --- | --- | --- |
| **Count component** | **IRR** | **p-value** | **IRR** | **p-value** |
| **Rural residence** | 1.64 (1.21, 2.20) | 0.001 | 1.63 (1.21, 2.19) | 0.001 |
| **Sex** (female vs. male) | 0.92 (0.79, 1.07) | 0.259 | 0.92 (0.79, 1.06) | 0.247 |
| **Distance from health centre**  (≥5 km vs. < 5 km) | 0.92 (0.78, 1.08) | 0.321 | 0.92 (0.79, 1.09) | 0.341 |
| **Thatched roof** | 1.11 (0.93, 1.32) | 0.25 | 1.09 (0.92, 1.30) | 0.306 |
| **SES** Least poor |  |  |  |  |
| Less poor | 1.51 (1.01, 2.24) | 0.044 | 1.53 (1.03, 2.27) | 0.036 |
| Poor | 1.71 (1.18, 2.49) | 0.005 | 1.72 (1.19, 2.50) | 0.004 |
| More poor | 1.68 (1.15, 2.46) | 0.008 | 1.71 (1.17, 2.50) | 0.006 |
| Most poor | 1.65 (1.14, 2.41) | 0.009 | 1.68 (1.15, 2.44) | 0.007 |
| **Antibody Response group** Low |  |  |  |  |
| Medium | 1.03 (0.84, 1.26) | 0.77 | 1.02 (0.83, 1.25) | 0.844 |
| High | 1.13 (0.92, 1.38) | 0.241 | 1.12 (0.92, 1.37) | 0.263 |
| **Bednet use** Low |  |  |  |  |
| Medium | 1.07 (0.87, 1.32) | 0.526 | 1.08 (0.88, 1.33) | 0.469 |
| High | 1.17 (0.95, 1.45) | 0.138 | 1.19 (0.97, 1.47) | 0.102 |
|  |  |  |  |  |
| **Binary component** | **OR** | **p-value** | **OR** | **p-value** |
| **Rural residence** | 0.25 (0.10, 0.58) | 0.001 | 0.28 (0.13, 0.58) | 0.001 |
| **Thatched roof** | 1.27 (0.51, 3.16) | 0.612 | 1.16 (0.53, 2.53) | 0.717 |
| **SES** Least poor |  |  |  |  |
| Less poor | 0.59 (0.23, 1.53) | 0.276 | 0.62 (0.25, 1.52) | 0.296 |
| Poor | 0.38 (0.14, 1.05) | 0.063 | 0.40 (0.16, 1.04) | 0.061 |
| More poor | 0.34 (0.11, 1.05) | 0.062 | 0.38 (0.14, 1.05) | 0.061 |
| Most poor | 0.07 (0.00, 1.67) | 0.101 | 0.12 (0.02, 0.74) | 0.022 |
| **Antibody Response group** Low |  |  |  |  |
| Medium | 1.28 (0.57, 2.87) | 0.549 | 1.21 (0.58, 2.54) | 0.607 |
| High | 1.02 (0.43, 2.44) | 0.964 | 1.00 (0.45, 2.20) | 0.997 |
| **Bednet use** Low |  |  |  |  |
| Medium | 0.86 (0.37, 1.98) | 0.723 | 0.90 (0.42, 1.93) | 0.795 |
| High | 0.54 (0.19, 1.52) | 0.244 | 0.62 (0.26, 1.50) | 0.287 |

AIC for ZINB model: 2444.9, for ZIPIG 2446.4; AIC for standard Poisson Inverse Gaussian (not shown) 2464.2. Vuong test of ZINB vs. standard negative binomial: z =3.02, P=0.0013, Vuong test of ZIPIG vs. standard Poisson inverse gaussian: z = -6.09 P = 1.0.

**References**

1. Hardin JW, Hilbe JM: *Generalized Linear Models and Extensions.* Third edn. College Station, Texas: Stata Press; 2012.

2. Hilbe JM: *Negative Binomial Regression.* Second Edition edn. Cambridge: Cambridge University Press; 2012.
